# Using alone at home: What’s missing in housing-based responses to the overdose crisis?

**DOI:** 10.1186/s12954-024-00933-y

**Published:** 2024-01-29

**Authors:** Taylor Fleming, Jade Boyd, Koharu Loulou Chayama, Kelly R. Knight, Ryan McNeil

**Affiliations:** 1 British Columbia Centre On Substance Use, 400-1045 Howe Street, Vancouver, BC V6Z 2A9 Canada; 2 Interdisciplinary Studies Graduate Program, University of British Columbia, 270-2357 Main Mall, Vancouver, BC V6T 1Z4 Canada; 3 Department of Medicine, University of British Columbia, St. Paul’s Hospital, 608-1081 Burrard Street, Vancouver, BC V6Z 1Y6 Canada; 4 Department of Humanities and Social Sciences, School of Medicine, University of California San Francisco, 490 Illinois Street, 7th Floor, San Francisco, CA 94143-0850 USA; 5 Department of Medicine, Yale School of Medicine, Yale University, 367 Cedar Street, New Haven, CT 10001 USA; 6 Program in Addiction Medicine, Yale School of Medicine, 367 Cedar Street, New Haven, CT 10001 USA

**Keywords:** People who use drugs, Overdose, Housing, Harm reduction interventions

## Abstract

**Background:**

Against the backdrop of North America’s overdose crisis, most overdose deaths are occurring in housing environments, largely due to individuals using drugs alone. Overdose deaths in cities remain concentrated in marginal housing environments (e.g., single-room occupancy housing, shelters), which are often the only forms of housing available to urban poor and drug-using communities. This commentary aims to highlight current housing-based overdose prevention interventions and to situate them within the broader environmental contexts of marginal housing. In doing so, we call attention to the need to better understand marginal housing as sites of overdose vulnerability and public health intervention to optimize responses to the overdose crisis.

**Harm reduction and overdose prevention in housing:**

In response to high overdose rates in marginal housing environments several interventions (e.g., housing-based supervised consumption rooms, peer-witnessed injection) have recently been implemented in select jurisdictions. However, even with the growing recognition of marginal housing as a key intervention site, housing-based interventions have yet to be scaled up in a meaningful way. Further, there have been persistent challenges to tailoring these approaches to address dynamics within housing environments. Thus, while it is critical to expand coverage of housing-based interventions across marginal housing environments, these interventions must also attend to the contextual drivers of risks in these settings to best foster enabling environments for harm reduction and maximize impacts.

**Conclusion:**

Emerging housing-focused interventions are designed to address key drivers of overdose risk (e.g., using alone, toxic drug supply). Yet, broader contextual factors (e.g., drug criminalization, housing quality, gender) are equally critical factors that shape how structurally vulnerable people who use drugs navigate and engage with harm reduction interventions. A more comprehensive understanding of these contextual factors within housing environments is needed to inform policy and programmatic interventions that are responsive to the needs of people who use drugs in these settings.

## Background

Responses to North America’s overdose crisis are increasingly centering harm reduction approaches, as interventions championed by community organizers are being integrated across public health systems. This includes implementation and scale-up of safer environment interventions (SEI; e.g., supervised consumption and overdose prevention sites), novel opioid agonist and maintenance therapies (e.g., oral and injectable hydromorphone), and other harm reduction services (e.g., drug checking, naloxone distribution) [1, 2]. Even as thousands of overdose deaths have been averted by these responses [3], the overdose crisis continues to worsen amidst rising polysubstance use and ongoing changes to the illicit drug supply, including fluctuations in the potency of fentanyl and fentanyl analogs and the emergence of new adulterants (e.g., xylazine, illicit benzodiazepines) [4]. A central challenge now facing North America’s overdose response is how to intervene to address these dynamics in the settings where the most overdose deaths occur: housing environments, which have been largely omitted from overdose prevention and response strategies to date. This is surprising; why has housing as a substantive overdose risk environment not been more prioritized as an important site of meaningful intervention?

In both the USA [5] and Canada [6] epidemiological data indicate that the majority of fatal overdoses occur in housing environments, largely due to individuals using criminalized drugs of unknown purity alone. Overdose deaths in cities tend to be concentrated in marginal housing environments [7], a term which refers to the collection of housing and shelter models that are often the only form of housing available to urban poor and drug-using communities. With some regional variation, the literature commonly captures certain forms of housing under this label, including shelters for homeless persons, single-room occupancy (SRO) housing, supportive housing, transitional housing, and social, welfare, or public housing [8–10]. Marginal housing environments are often characterized by, for example, poor housing quality, housing insecurity, stigma, and neighborhood disadvantage.

In British Columbia (BC) over 80% of overdose deaths occur in housing and, while most overdoses occur in private residences across the province, almost 50% of fatal overdoses in the Vancouver region are occurring in marginal housing environments [11]. Similar patterns in the distribution of fatal overdoses have been observed in the USA, further underscoring the relationship between marginal housing and overdose risk. For example, Rowe et al. [7] found the overdose mortality rate among residents of SRO housing in San Francisco was almost 20 times higher than that of non-SRO residents, with 86% of overdose deaths among SRO residents occurring in their own unit. A New York-based study found that over half of fatal overdoses among recently housed persons occurred in supportive housing [12]. These overdoses are also more likely to result in death than those occurring outside of one’s own housing [13]. Such environments thus represent a key site for overdose risk and intervention. However, overdose prevention interventions and accompanying research have mainly focused on individuals using in settings where formal (e.g., supervised consumption sites) or informal (e.g., community naloxone distribution) supervision is possible [2]. As a result, marginal housing remains poorly understood as an overdose risk environment and site of potential intervention, with much of the policy and research communities’ attention remaining focused on community-based SEIs (e.g., overdose prevention sites, naloxone distribution) that are not necessarily tailored to specific dynamics of housing environments. This persists even as research has pointed to the need to rapidly extend SEIs and other harm reduction interventions into housing environments [14].

Researchers have increasingly focused attention on how marginal housing functions as a risk environment, detailing how the environmental contexts of these spaces can produce a range of health and social harms including violence, poor mental health outcomes, negative HIV outcomes, and risks stemming from drug criminalization [15–17]. Attention to broader environmental forces allows us to contextualize the disproportionate burden of overdose vulnerability in these settings by highlighting how a range of environmental forces intersect to produce overdose-related risks: socio-cultural forces (e.g., drug-related stigma and discrimination, gendered and racialized violence); structural forces at macro- (e.g., drug criminalization, policing strategies) and microlevels (e.g., building-specific guest and anti-drug policies); and physical characteristics of marginal housing environments (e.g., poor housing quality, pest infestations, surveillance technologies). Taken together, these dynamics can, for example, lead people to use drugs alone to conceal their drug use to avoid eviction and possible contact with police [18].

Further, certain groups of people who use drugs (PWUD) are differentially vulnerable to harm in these environments dues to the ways in which their social positions within larger networks of power shape and constrain agency [19]. Applying this "structural vulnerability" lens reveals how poor health and social outcomes are produced in response to social–structural drivers (e.g., extreme poverty, housing insecurity, drug criminalization) and multiple interesting oppressions (e.g., racism, sexism), as opposed to individual choices. For example, the largely men-centered models under which marginal housing typically operates have been implicated in experiences of violence, sexual and economic exploitation, and drug-related risks, including overdose vulnerability among women, transgender, and Two-Spirit persons [15–17]. Moreover, this form of housing is generally concentrated in socio-economically marginalized neighborhoods, which experience greater housing vulnerability, political–economic neglect, and gentrification pressures, and are the site of significant—and often racialized—health disparities [20].

Marginal housing environments are also sites of intensive social control (e.g., via surveillance technologies, prohibitive building policies, and policing presence) that place limitations on autonomy and identity, with the narratives of residents often positioning them more as institutional settings akin to prisons than housing [9, 21]. This is significant as overregulation has been associated with social withdrawal and isolation in housing, specifically [21], and socio-economically marginalized communities, more broadly [22]. Research on supervised consumption sites (SCS) has documented how surveillance and control mechanisms embedded in these spaces impact socio-spatial drug use practices and can serve as a barrier to access by positioning SCS as sites of specific risks (e.g., policing, stigma), rather than harm reduction settings [23]. This raises questions regarding how novel overdose prevention and response interventions can be implemented within housing environments against this backdrop to reshape environmental contexts of drug use without reproducing forms of oppression.

## Harm reduction and overdose prevention in housing

In response to high overdose rates in marginal housing environments, housing-based overdose prevention and response interventions (e.g., housing-based supervised consumption rooms, peer-witnessed injection) are being implemented in some jurisdictions [15, 24, 25]. Such interventions attempt to extend the impacts of SEIs from community- and clinic-based settings into housing environments. However, even with the growing recognition of marginal housing environments as a key intervention site, housing-based overdose prevention interventions have yet to be scaled up in a meaningful way. Further, there have been persistent challenges to tailoring these approaches to address dynamics within housing environments.

On a more immediate level, the coverage of overdose prevention and response interventions in housing environments remains inadequate. For example, medical health officers in BC can declare a housing site as a temporary overdose prevention site (OPS) as part of the province’s pandemic response. However, these sites remain rare, with authorities instead encouraging expansion of community distribution of take-home naloxone and drug testing strips rather than explicit housing-based services [26]. In the USA, housing-based models are in their infancy—a peer-based model was recently implemented in San Francisco, and there are plans to pilot overdose prevention strategies in supportive housing in New York State [25, 27].

Provision of harm reduction services has not previously been a priority in housing environments, even as Housing First models have been embraced and targeted toward PWUD in settings across the USA and Canada. While Housing First is based on a harm reduction model, this has not translated into the meaningful extension of harm reduction approaches into housing environments, with two recent reviews finding an absence of harm reduction, either in practice or discussion, in the Housing First literature [28, 29]. Moreover, research has found disagreement between the higher level of support services purported to be offered within a Los Angeles supportive housing program and those readily available to residents [30], further highlighting the discord between harm reduction policies and practices. Abstinence requirements and policies governing on-site drug use further complicate understandings of harm reduction in many housing environments and underscore overdose risk. Pauly, Wallace, and Barber [31] found that harm reduction practices in an abstinence-only transitional housing program consisted of staff “*turning a blind eye*” to drug use, thereby inadvertently encouraging riskier drug use practices (p. 24). Additionally, housing-based harm reduction has long been limited to distributions of syringes, pipes, and more recently, naloxone, with limited interventions available to address overdose risk [32]. While an important part of SEIs, supply distribution alone is an incomplete response—any housing-based interventions must address the full spectrum of drug-related harms.

Due to the lack of scale up of housing-based interventions, there is a paucity of available research on harm reduction and overdose prevention strategies explicitly targeting vulnerably housed PWUD. SCS/OPS have been extended into select housing environments with mixed results. While they have a robust evidence base demonstrating effectiveness in mitigating overdose vulnerability [3], as place-based interventions, SCS/OPS cannot be directly translated to housing environments without accounting for the specific contextual forces operating within these spaces. Collins et al.’s [15] research examining women’s use of housing-based OPS in Vancouver SROs found that the social–structural features of these environments, including fears of gender-based violence and restrictions on inhalation drug use, served as a barrier to access as women felt they were safer when using alone. This study also highlighted how the effectiveness of housing-based OPS can be limited when the risks of using alone are viewed as less pressing due to other concerns (e.g., violence) and perceived ability to manage overdose risk oneself. Conversely, research undertaken at a women-only transitional housing site elsewhere in the Vancouver area demonstrated favorable perceptions and high usage of the on-site SCS, which included a sanctioned smoking space [33]. Researchers credited this to the inclusion of a smoking space—most participants preferred to smoke their drugs—and how the women-only environment limited gendered violence and fostered a social environment that promoted mutual support (e.g., drug sharing) and harm reduction practices (e.g., overdose response). This highlights the further need for interventions and subsequent research that consider the unique environmental contexts of marginal housing spaces, and how these broader forces impact drug use practices and, thus, overdose vulnerability.

Peer-based models, which are considered a key component of successful harm reduction interventions [34], have also received limited attention in the housing and harm reduction literature. A peer-witnessed supervised injection intervention implemented in two emergency shelters was found to be effective in facilitating overdose response due to notions of care, trust, and solidarity embedded within the social dynamics of drug-using communities [35]. Programs in Vancouver, Toronto, and San Francisco that trained dedicated tenant overdose responders in select marginal housing environments found similar results regarding the strengths of peer-based responses in engaging PWUD in harm reduction, and mutual care more broadly, as well as demonstrated the organizing potential in these spaces [18, 24, 25, 36]. However, these studies also suggest that social–structural contexts of marginal housing environments can act as a barrier to implementation due to, for example, fears of landlord retaliation or prejudicial treatment based on drug use. This underscores the critical need for alignment between building management and programmatic harm reduction goals. Furthermore, the lack of resources made available for peer-based responses (e.g., no or inadequate financial compensation, lack of emotional supports) adversely impacted their sustainability.

Overdose monitoring technologies have also been considered specifically for individuals using drugs alone in housing, including smartphone apps and button-alert systems [37], although these have not been without challenges. For example, smartphone apps may be an effective overdose monitoring technology for PWUD experiencing greater socioeconomic and housing stability [37, 38]. However, they are often poorly aligned with the lived realities of structurally vulnerable PWUD, who often experience unstable housing and inconsistent cellphone access [38]. There is also evidence that overdose monitoring technologies, like button-alert systems, might not be as responsive as hoped to overdose vulnerability when implemented in real-world settings. In one study, researchers in Vancouver evaluated a button alert system installed in the rooms of a women-only SRO, which was meant to mitigate the risks associated with using drugs alone [39]. With this intervention individuals could press a wall-mounted button before using drugs alone in their room, which would alert staff to check on them and respond accordingly within a set amount of time. However, researchers found that women primarily used the system to alert staff of emergencies in progress (e.g., gender-based violence, other resident actively overdosing) rather than its intended purpose of overdose prevention. Conversely, preliminary reporting from a pilot of the same button alert system implemented in a San Francisco SRO suggests they provided a proactive method for harm reduction, greater privacy during emergencies, as well as other beneficial uses in addition to overdose response (e.g., calling for help in situations of violence or when a visitor would not leave) [25]. Together these projects demonstrated important and positive, if not alternative, uses for the technology that provide a valuable compliment to other housing-based harm reduction programming (e.g., peer-based responses, naloxone distribution) [25, 39]. However, they also highlight the need for additional interventions that target the immediate risks associated with using drugs alone, particularly in the context of a toxic drug supply. Furthermore, overdose monitoring technologies continue the pattern of individualizing responsibility for overdose risk management among structurally vulnerable groups—in this case, PWUD living in marginal housing environments—through behavioral interventions rather than addressing systemic drivers of risks.

## Looking ahead: implications for research and practice

Even as marginal housing environments are positioned as “safe havens” when compared to homelessness, they remain risk environments that require contextually informed interventions that are responsive to their social, structural, and physical conditions. The social, structural, and physical characteristics of these spaces that mark them as risk environments also complicate their potential to act as enabling environments for overdose risk reduction. However, spaces of risk and harm can be remade into spaces of safety and well-being through thoughtful, drug user-led intervention [36], and marginal housing need not be fated to be an overdose risk environment. We can see a pathway for how housing-focused interventions can be made responsive to both the key environmental drivers of overdose vulnerability in marginal housing *and* the evolving risks associated with the toxic drug supply, and thus may be repositioned as spaces of reduced risk. However, there remain numerous harms embedded within the routine functions of marginal housing environments that will continue to produce vulnerability to overdose and other harms if unaddressed. Thus, while it is critical to expand coverage of housing-based interventions across marginal housing environments, the social, structural, and environmental drivers of risks in these settings (e.g., guest policies, drug-related evictions) must be attended to in order to best foster enabling environments for harm reduction and maximize the impacts of harm reduction interventions. This includes, for example, strengthening tenancy protections to increase housing security and reduce risks of retaliatory evictions, reassessing building-specific regulations (e.g., guest policies, curfews) to balance concerns related to safety with resident autonomy, and better aligning operational contexts with harm reduction goals (e.g., supporting safer inhalation).

Further, public health messaging recommending against using alone discounts the numerous risks mitigated by this practice (e.g., social and gendered violence, police involvement, rushed injections, sharing equipment) that cannot necessarily be accounted for even in spaces intended for PWUD (e.g., SCS, housing OPS) [40]. It is also important to acknowledge that increased comfort and pleasure from using drugs alone is a valid reason that many PWUD cite [41]. Just as research is increasingly acknowledging the role of pleasure in drug use, so too must pleasure factor into our understandings of specific drug use practices. As such, there is a need for research that accommodates and accounts for pleasure within the context of using alone at home to better inform housing-based interventions. More broadly, innovations in housing-based harm reduction must be more pragmatic in their approach and consider how using alone can be repositioned as both a risk mitigation and pleasurable practice within the context of structural vulnerability. Peer-based responses and overdose monitoring technologies have demonstrated potential in this regard [25, 36] and should have greater resources allocated toward them to support sustainability and advance equity-related goals. However, addressing the unpredictable drug supply is among the most impactful means to reduce overdose risk for PWUD using alone in housing.

Early research on delivery of a safer supply (e.g., prescribed hydromorphone) program in a supportive housing space is promising, proposing that access to regulated pharmaceutical grade medications can mediate the relationship between these spaces and overdose risk by addressing broader social–structural contexts of criminalized drug use and housing vulnerability [42]. While this work used secondary data and was not designed to explore the impact of safer supply on overdose risk in marginal housing, it suggests the need for dedicated research on this phenomenon, particularly given that most safer supply programs are community- or clinic-based. Thus, as more novel interventions to address the toxic drug supply are implemented (e.g., drug testing technologies, safer supply) questions emerge regarding their impact on how PWUD negotiate risks in housing environments.

Building on the historical leadership of drug-user activists and community organizers in championing innovative and evidence-based responses to drug-related risks (e.g., syringe distribution, SCS/OPS), it is critical that any approaches to address overdose risk in marginal housing meaningfully involve PWUD living in these environments. Harm reduction models that center people with lived experience throughout planning and implementation have proven successful in establishing interventions that are more responsive to the needs of PWUD, and can be considered best practice within community-based harm reduction frameworks [34]. We have seen this approach applied in development of a peer-based overdose response intervention in Vancouver’s marginal housing, wherein the care and mutual support fostered by the peer-led nature of the program was reported to have cascading impacts beyond reducing drug-related risks, including community-building, housing advocacy, and helping people “*stand up and have a voice*” [36, p. 5]. Future innovations in housing-based harm reduction and overdose prevention should be guided by PWUD with lived/living experience in marginal housing so as not to reproduce the limitations of past and current interventions, and to facilitate programming that is responsive to marginal housing environments as a unique context.

## Conclusion

Overall, despite recent advancements in the implementation of harm reduction interventions in North America, the attention to housing as an intervention site from policy and research communities remains disproportionate to the degree of overdose risk present in these environments. Emerging housing-focused interventions may appear to address key drivers of overdose risk for structurally vulnerable PWUD living in marginal housing by reshaping the environmental contexts of drug use to support safer drug use practices, primarily through some form of supervision. However, broader contextual factors (e.g., drug criminalization, housing quality, racialized and gender-based violence) are critical in shaping how PWUD navigate and engage with harm reduction interventions across settings, and must be considered within unique environmental contexts to optimize the effectiveness of overdose response. That SEIs have not been successfully scaled in marginal housing suggests the need to critically examine how the social, structural, and physical environments of these spaces produce overdose vulnerability, and to inform policy and programmatic interventions that are responsive to the structural vulnerabilities of PWUD in housing contexts.

## Data Availability

Not applicable.
